# Methodological quality of clinical practice guidelines with physical activity recommendations for people diagnosed with cancer: A systematic critical appraisal using the AGREE II tool

**DOI:** 10.1371/journal.pone.0214846

**Published:** 2019-04-10

**Authors:** Shirin M. Shallwani, Judy King, Roanne Thomas, Odette Thevenot, Gino De Angelis, Ala’ S. Aburub, Lucie Brosseau

**Affiliations:** 1 School of Rehabilitation Sciences, Faculty of Health Sciences, University of Ottawa, Ottawa, Ontario, Canada; 2 Lymphedema Program & Physiotherapy Department, McGill University Health Centre, Montreal, Quebec, Canada; 3 Physical Therapy Department, Faculty of Allied Medical Sciences, Isra University, Amman, Jordan; Aix-Marseille Universite, FRANCE

## Abstract

Evidence suggests physical activity (PA) is beneficial for people diagnosed with cancer. Clinical practice guidelines provide specific recommendations based on available research and are useful in informing evidence-based practice and guiding future research. Little is known on the extent and quality of guidelines on PA targeted to the cancer population. The objectives of this systematic review were to: 1) identify recent clinical practice guidelines including PA or exercise recommendations for people with cancer and 2) critically appraise the methodological quality of the included guidelines. A systematic search of four electronic databases (MEDLINE, EMBASE, CINAHL and PEDro) and supplementary sources was conducted. Two reviewers independently scanned articles and selected guidelines for inclusion according to the following criteria: published in English, developed or updated in previous five years (January 2012-June 2017), published in peer-reviewed scientific journals, including ≥1 specific recommendation on PA or exercise, and relevant to adults diagnosed with cancer. Subsequently, two trained assessors independently appraised the included guidelines using the Appraisal of Guidelines for Research and Evaluation (AGREE) II tool. Average scores for six domains (scope and purpose; stakeholder involvement; rigour of development; clarity of presentation; applicability; and editorial independence) and overall quality were calculated. From the literature search, we identified 29 articles, representing 20 sets of guidelines meeting the selection criteria. The guidelines were applicable to the following cancer populations: general (n = 9), breast (n = 5), lung (n = 2), colorectal (n = 1), head and neck (n = 1), myeloma (n = 1) and prostate (n = 1). The guidelines were generally of moderate methodological quality (mean AGREE II overall quality score: 4.6/7, range 2.5–6). The area of lowest quality was in the domain of applicability (mean AGREE II quality domain score: 40%), whereas the strongest domains were related to scope and purpose (81%) and clarity of presentation (77%). Although there are limitations in the primary research informing the recommendations, guidelines of acceptable quality exist to direct stakeholders on targeted PA recommendations for a range of cancer populations. Improvement is needed in the applicability of guidelines to enhance their relevance and clinical use. Health professionals can play an important role in supporting people with cancer throughout the disease trajectory and benefit from access to well-developed and appropriate materials to interpret research knowledge on effective rehabilitation strategies, including PA.

## Introduction

In the United States of America, it is estimated that 1.7 million individuals were diagnosed with cancer in 2017 and the lifetime probability of developing cancer is around 40% in men and women [1]. With advances in early detection and treatment options, improved survival rates after diagnosis have been found across most cancer types over time [1]. An individual is considered to be a cancer survivor “from the time of diagnosis until the end of life” [2]. Cancer and its treatments can have debilitating impacts on survivors and their wellbeing both in the short-term and long-term. Chronic issues associated with cancer include persistent fatigue, muscle stiffness and joint pain [3], cardiopulmonary dysfunction [4], difficulty with mobility and daily activities [3], and psychological effects, such as depression, anxiety and post-traumatic stress disorder [3].

There is evidence demonstrating physical activity (PA), traditionally defined as “any bodily movement produced by skeletal muscles that results in energy expenditure” [5], can mitigate certain side effects of cancer and its treatments. In meta-analyses of controlled studies, reported benefits with structured exercise interventions for individuals during and post-treatment for cancer include reduced fatigue, enhanced physical function, improved psychosocial status and fewer treatment complications [6, 7]. In a review of observational studies, PA was found to be associated with decreased all-cause, breast cancer–specific, and colon cancer–specific mortality [8]. These findings are promising and warrant further exploration of PA programs that are evidence-based and tailored for people diagnosed with cancer.

Clinical practice guidelines are defined as “systematically developed statements to assist practitioner and patient decisions about appropriate healthcare for specific clinical circumstances” [9]. Relevant guidelines may be valuable in guiding cancer survivors, caregivers and health professionals on appropriate PA after cancer diagnosis and informing the development and implementation of evidence-based interventions in clinical practice. Moreover, these knowledge tools are useful in identifying gaps in the evidence base and guiding future research.

In their review, Buffart et al. (2014) identified PA guidelines for cancer survivors and highlighted the generic nature of recommendations for this population. They emphasized the need for tailored, specific recommendations targeted to individuals with cancer [10]. This review was limited to guidelines focusing on PA. Harris et al, (2012) identified and appraised guidelines on breast cancer rehabilitation and presented specific recommendations for the management of particular impairments, such as pain, fatigue and lymphedema [11]. While several high-quality guidelines were identified, this review addressed the general rehabilitation management of the breast cancer population and did not specifically focus on PA prescription. In this review, the need for enhanced quality and frequent updating of pertinent guidelines was highlighted [11]. Limitations in the research base informing guidelines in these areas have been noted, particularly with respect to identifying optimal dosage parameters, mode and timing for exercise interventions, evaluating the effect of PA on other outcomes, such as arthralgia and sarcopenia, and testing exercise interventions in less common cancer subgroups [10–13]. Over the last five years, the emergence of a growing body of evidence in this area [14] urgently highlights the need for specific, targeted and updated recommendations on PA for individuals diagnosed with cancer.

Research suggests PA can be beneficial in optimizing physical functioning and enhancing quality of life in people diagnosed with cancer within different phases of the disease trajectory. The development of high-quality knowledge tools (e.g. practice guidelines) and the translation of this knowledge to clinical practice are urgently needed. To our knowledge, little is known about the extent and quality of recent guidelines with PA recommendations for the cancer population. The recognition of methodological limitations within existing guidelines and the identification of relevant recommendations supported by robust evidence will promote high-quality research and facilitate knowledge translation strategies in the field of cancer rehabilitation. Furthermore, this information will contribute to the scientific knowledge on PA as a potentially effective rehabilitation strategy for the cancer population and highlight gaps in the current evidence base. The objectives of this systematic review were to identify recent clinical practice guidelines including PA or exercise recommendations targeted to individuals diagnosed with cancer and to critically appraise the methodological quality of the included guidelines.

## Materials and methods

### Part 1: Systematic review of the literature

#### Search strategy

The literature was systematically reviewed to identify recent clinical practice guidelines with PA recommendations for individuals diagnosed with cancer. Search strategies were developed by the study researchers and reviewed by a librarian (MCD) at the University of Ottawa Health Sciences Library. The search strategies are provided in S1 Table. In June 2017, searches were conducted in the following electronic databases: Medical Literature Analysis and Retrieval System Online (MEDLINE), Excerpta Medica Database (EMBASE), Cumulative Index to Nursing and Allied Health Literature (CINAHL) and Physiotherapy Evidence Database (PEDro). Subsequently, in December 2017, supplementary searches were conducted by hand searching reference lists of key articles and relevant conference abstracts as well as performing searches within the electronic databases of the National Guideline Clearinghouse, the National Institute for Health and Care Excellence and the Canadian Medical Association Infobase.

#### Article selection

Using the EndNote X7.8 software (Clarivate Analytics, PA, USA), the primary reviewer (SMS) performed an initial screening of titles to remove irrelevant articles. The remaining articles were scanned at the abstract level and subsequently at the full-text level by two independent reviewers (GDA and SMS). Relevant articles were selected according to pre-defined selection criteria, detailed in S2 Table. Clinical practice guidelines were included if they were published in English, recently developed or updated in the previous five years (January 2012 to June 2017), published in peer-reviewed scientific journals and provided specific recommendations on at least one PA or exercise parameter (frequency, intensity, duration and/or type) for adult (aged ≥18 years) cancer survivors, defined as individuals at any time point following cancer diagnosis [2]. In cases where guidelines were recently updated, previously published guidelines were reviewed to obtain relevant details related to methodology. All study outcomes were included in this review. Discrepancies in article selection were resolved through discussion or consultation with a third senior reviewer (LB) to achieve consensus.

### Part 2: Critical appraisal of guidelines

#### Instrument

The Appraisal of Guidelines for Research and Evaluation **(**AGREE II) tool is a validated and reliable instrument to appraise the methodological quality of guidelines [15, 16]. It includes 23 items each scored on a 7-point scale, comprising six domains: 1) scope and purpose; 2) stakeholder involvement; 3) rigour of development; 4) clarity of presentation; 5) applicability; and 6) editorial independence.

#### Training of appraisers

Two appraisers (SMS and OT) received training on the use of the AGREE II tool with a senior appraiser (PR) and the online tutorial [17]. Prior to this review, the appraisers evaluated a guideline on osteoarthritis using the AGREE II tool to familiarize themselves with the instrument and compare their scores.

#### Appraisal of guidelines

The two appraisers referred to the AGREE II instrument with the user’s manual [18] and independently evaluated each guideline included in the review. A grade was assigned for each of the 23 AGREE II items on a 7-point scale, with a score of 1 indicating the item met none of the criteria or was very poorly reported and a score of 7 indicating the item met all the criteria and was well reported. For each guideline, the appraisers also assigned an overall quality assessment score using the same 7-point scale. Any major discrepancies (≥3 points difference) in AGREE II item scores were resolved by discussion between the two appraisers.

#### Calculation of domain scores

The AGREE II item ratings were entered into the AGREE II score concordance calculator developed by the Capacity Enhancement Program at McMaster University [19, 20] to determine scaled scores for each domain. Domain quality scores were calculated by summing the item scores in a given domain and converting the number into a standardized percentage of the maximum score that can be obtained for that domain:

Scaleddomainscore=(obtainedscore‑minimumpossiblescore)/(maximumpossiblescore)

Maximumpossiblescore=7(stronglyagree)x23itemsx2appraisers

Minimumpossiblescore=1(stronglyagree)x23itemsx2appraisers

#### Interpretation of domain scores

The AGREE II consortium has not set specific cut-off scores to differentiate between high and low-quality guidelines. For this review, a guideline of acceptable quality required a score of at least 60% for rigour of development (domain 3) as well as 60% in at least two other domains. This quality threshold was based on cut-off scores reported in previous guideline appraisals [20–22].

## Results

### Search results

**Fig 1:**
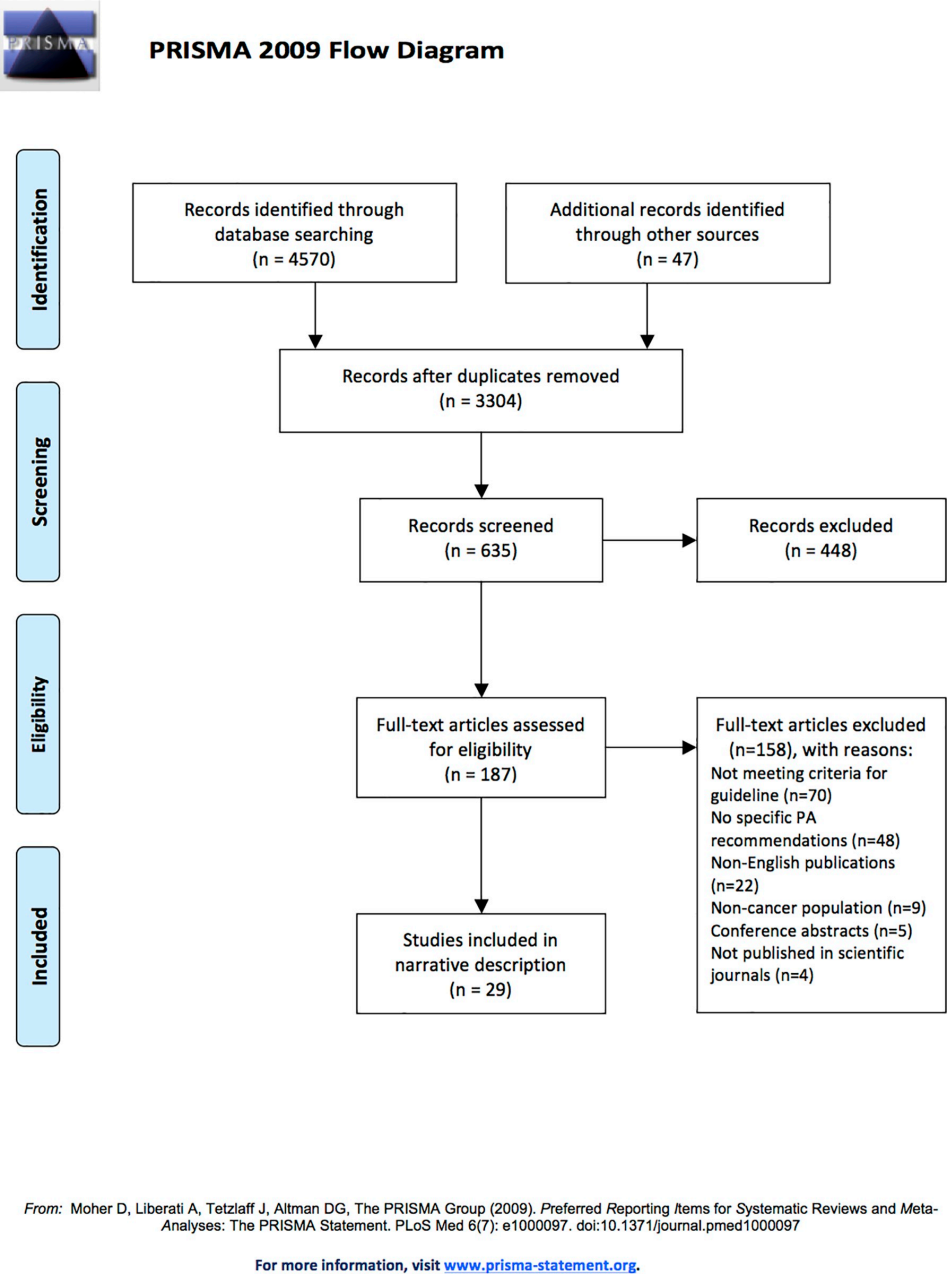
PRISMA flow diagram.

The initial electronic database search yielded 4570 articles, whereas the search of supplementary sources resulted in an additional 47 results (Fig 1). After the removal of duplicates and two rounds of article screening at the title level (n = 3304) and abstract level (n = 635), there were 187 full texts reviewed. Reasons for exclusion (n = 158) are provided in Fig 1. A list of excluded articles may be obtained from the corresponding author.

### Description of included guidelines

From the literature search, we identified 29 articles, representing 20 sets of guidelines (Table 1). Half of the guidelines were published by expert teams based in the USA (n = 10, 50%). Guidelines were mostly applicable to the general (n = 9) or breast (n = 5) cancer populations. Fewer guidelines were targeted to people diagnosed with lung (n = 2), colorectal (n = 1), head and neck (n = 1), myeloma (n = 1) and prostate (n = 1) cancers.

**Table 1:** Description of included guidelines with PA recommendations for individuals diagnosed with cancer.

	Guideline	Organization (country)	Title	Topic
General cancer (n = 9)				
**1.**	Almeida et al. (2012) [23]	ABMFR (Brazil)	Exercise for oncological patients: rehabilitation	Exercise
**2.**	Arends et al. (2017) [24]	ESPEN (International)	ESPEN guidelines on nutrition in cancer patients	Nutrition
**3.**	Berger et al. (2015) [25]	NCCN (USA)	Cancer-related fatigue, version 2.2015	Fatigue management
**4.**	Bower et al. (2014) [26]	ASCO (USA)	Screening, assessment, and management of fatigue in adult survivors of cancer: an ASCO clinical practice guideline adaptation	Fatigue management
**5.**	Denlinger et al., (2014) [27–32]	NCCN (USA)	Survivorship: healthy lifestyles, version 2.2014	Survivorship care Healthy lifestyles
			Survivorship: nutrition and weight management, version 2.2014	Nutrition
			Survivorship: cognitive function, version 1.2014	Cognitive function
			Survivorship: pain version 1.2014	Pain management
			Survivorship: sleep disorders, version 1.2014	Sleep disorders
			Survivorship: fatigue, version 1.2014	Fatigue management
**6.**	Howell et al., (2013) [33]	N/A (Canada)	A pan-Canadian practice guideline and algorithm: screening, assessment, and supportive care of adults with cancer-related fatigue	Fatigue management
**7.**	Paice et al. (2016) [34]	ASCO (USA)	Management of chronic pain in survivors of adult cancers: ASCO clinical practice guideline	Pain management
**8.**	Rock et al. (2012) [35]	ACS (USA)	Nutrition and physical activity guidelines for cancer survivors	Nutrition and physical activity
**9.**	Segal et al. (2017) [36]	CCO (Canada)	Exercise for people with cancer: a clinical practice guideline	Physical activity
**Breast cancer (n = 5)**				
**1.**	Brito et al. (2012) [37]	ABMFR (Brazil)	Breast cancer: rehabilitation	Rehabilitation
**2.**	Greenlee et al. (2017) [38, 39]	SIO (International)	Clinical practice guidelines on the evidence-based use of integrative therapies during and after breast cancer treatment	Integrative therapies
**3.**	Hadji et al. (2017) [40]	Multiple (International)	Management of aromatase inhibitor-associated bone loss (AIBL) in postmenopausal women with hormone sensitive breast cancer: joint position statement of the IOF, CABS, ECTS, IEG, ESCEO IMS, and SIOG	Bone loss
**4.**	Runowicz et al. (2016) [41, 42]	ACS / ASCO (USA)	American Cancer Society/American Society of Clinical Oncology breast cancer survivorship care guideline	Survivorship care
**5.**	Witt & Cardoso (2016) [43]	N/A (International)	Complementary and integrative medicine for breast cancer patients—Eevidence based practical recommendations	Integrative therapies
**Other cancers (n = 6)**				
**1.**	El-Shami et al. (2015) [44]	ACS (USA)	American Cancer Society colorectal cancer survivorship care guidelines	Survivorship care
**2.**	Cohen et al. (2016) [45]	ACS (USA)	American Cancer Society head and neck cancer survivorship care Guideline	Survivorship care
**3.**	Cecatto et al. (2012) [46]	ABMFR (Brazil)	Lung neoplasms: rehabilitation	Rehabilitation
**4.**	Deng et al. (2013) [47]	ACCP (USA)	Complementary therapies and integrative medicine in lung cancer: diagnosis and management of lung cancer, 3rd ed: American College of Chest Physicians evidence-based clinical practice guidelines	Integrative therapies
	Detterbeck et al. (2013) [48]		Executive summary: diagnosis and management of lung cancer, 3rd ed: American College of Chest Physicians evidence-based clinical practice guidelines	
**5.**	Snowden et al. (2017) [49]	BSH (UK)	Guidelines for screening and management of late and long-term consequences of myeloma and its treatment	Myeloma
**6.**	Skolarus et al. (2014) [50]	ACS (USA)	American Cancer Society prostate cancer survivorship care guidelines	Survivorship care

ACS: American Cancer Society, ACCP: American College of Chest Physicians, ASCO: American Society of Clinical Oncology, ABMFR: Brazilian Association of Physical Medicine and Rehabilitation, BSH: British Society for Haematology, CCO: Cancer Care Ontario, ESPEN: European Society for Clinical Nutrition and Metabolism, NCCN: National Cancer Comprehensive Network, SIO: Society for Integrative Oncology

### Methodological quality of guidelines

For each guideline included in this review, the AGREE II domain scores are presented in Table 2. The guidelines were generally of moderate methodological quality (mean AGREE II overall quality score: 4.6/7, range 2.5–6). The domain of applicability had the lowest mean quality score (40%), whereas the domains of highest quality were scope and purpose (81%) and clarity of presentation (77%).

**Table 2:** AGREE II domain and overall quality scores of included guidelines.

**AGREE II Domain**	**1. Scope & Purpose**	**2. Stakeholder Involvement**	**3. Rigour of Development**	**4. Clarity of Presentation**	**5. Applicability**	**6. Editorial Independence**	**Overall Quality Score**
**General cancer**							
Almeida et al. (2012) [23]	78%	17%	38%	47%	8%	21%	2.5/7
Arends et al. (2017) [24]	89%	83%	83%	72%	75%	83%	5.5/7
Berger et al. (2015) [25]	86%	58%	34%	53%	29%	79%	4.5/7
Bower et al. (2014) [26]	92%	86%	65%	92%	44%	92%	5/7
Denlinger et al., (2014) [27–32]	89%	78%	49%	100%	75%	83%	5.5/7
Howell et al., (2013) [33]	75%	69%	77%	86%	31%	50%	4.5/7
Paice et al. (2016) [34]	89%	89%	91%	94%	54%	88%	6/7
Rock et al. (2012) [35]	83%	72%	26%	61%	29%	54%	4/7
Segal et al. (2017) [36]	97%	69%	76%	83%	35%	92%	5/7
**Breast cancer**							
Brito et al. (2012) [37]	56%	17%	43%	50%	19%	25%	3.5/7
Greenlee et al. (2017) [38, 39]	69%	72%	89%	64%	29%	67%	5/7
Hadji et al. (2017) [40]	56%	42%	42%	47%	25%	42%	2.5/7
Runowicz et al. (2016) [41, 42]	83%	75%	78%	86%	50%	88%	5/7
Witt et al. (2016) [43]	72%	33%	46%	92%	54%	67%	4.5/7
**Colorectal cancer**							
El-Shami et al. (2015) [44]	92%	67%	85%	92%	54%	83%	5/7
**Head and neck cancer**							
Cohen et al. (2016) [45]	86%	83%	80%	92%	42%	83%	5/7
**Lung cancer**							
Cecatto et al. (2012) [46]	83%	22%	36%	69%	15%	21%	3/7
Deng et al. (2013) [47, 48, 51]	83%	83%	89%	86%	60%	96%	6/7
**Myeloma**							
Snowden et al. (2017) [49]	83%	83%	72%	89%	40%	79%	5/7
**Prostate cancer**							
Skolarus et al. (2014) [50]	81%	72%	83%	86%	25%	54%	5/7
**Mean scores**	**81%**	**64%**	**64%**	**77%**	**40%**	**67%**	**4.6/7**

#### Domain 1: Scope and purpose

For the 20 sets of guidelines, the mean AGREE II score for the domain of scope and purpose was 81%. Most of the included guidelines exceeded the quality threshold of 60% for this particular domain (n = 18, 90%). The guidelines receiving lower scores were generally due to an unclear description of characteristics related to the target population, such as disease stage and treatment phase.

#### Domain 2: Stakeholder involvement

The mean quality score for the domain of stakeholder involvement was 64% for the included guidelines. Of the 20 sets of guidelines, over two-thirds met the quality threshold for this domain (n = 14, 70%). The guidelines generally scored lower in this domain for several reasons, including missing relevant stakeholders and professionals from their research teams, particularly patient representatives.

#### Domain 3: Rigour of development

The included guidelines had a mean score of 64% for the section on rigour of development. In particular, three guidelines by Deng et al. (2013) [47], Greenlee et al. (2017) [39] and Paice et al. (2016) [34] scored exceptionally high in this domain (>85%). Items where several guidelines scored poorly included utilizing systematic methods to search the literature and formulate the recommendations, obtaining external review of the recommendations and considering a process to update the reports with new evidence.

#### Domain 4: Clarity of presentation

On the domain of clarity of presentation, the guidelines obtained a mean AGREE II score of 77%. Many of the guidelines reached the quality threshold for this domain (n = 16, 80%). Lower item scores were generally assigned when different options or conditions for management were not appropriately presented.

#### Domain 5: Applicability

For the included guidelines, the mean quality score for the domain of applicability was 40%. Besides Arends et al. (2017) [24], Deng et al. (2013) [47] and Denlinger et al. (2014) [27–32], none of the guidelines met the quality threshold for this domain. AGREE II items frequently receiving low scores in the included guidelines included the consideration of resource implications and the presentations of auditing or monitoring criteria.

#### Domain 6: Editorial independence

For editorial independence, the mean AGREE II domain score of the guidelines was 67%. Major funding sources and related conflicts of interest were acknowledged in most articles. Few guidelines explicitly stated that the funding source did not influence the content of the guideline.

#### Quality threshold

Over half of the guidelines (n = 12, 60%,) met the quality threshold with domain scores of ≥60% for rigour of development and at least two other domains (Table 2). These guidelines had a mean overall quality score of 5.2/7 (range 4.5–6). Five of these guidelines focused on the general cancer population [24, 26, 33, 34, 36] and covered topics including symptom management (n = 3), nutrition (n = 1) and exercise (n = 1). The remaining seven guidelines focused on specific cancer populations [39, 41, 44, 45, 47, 49, 50] and topics ranged from survivorship care (n = 4), complementary and integrative therapies (n = 2) and management of late / long-term effects (n = 1).

## Discussion

To our knowledge, this review provides the first systematic critical appraisal of guidelines with PA recommendations for individuals with cancer that were recently published in peer-reviewed scientific journals. In this review, 20 set of guidelines published from January 2012 to June 2017 including at least one specific PA recommendation targeted to people at any point after diagnosis of cancer were identified. The majority of guidelines (70%) were applicable to the general or breast cancer populations. These findings demonstrate the need for better developed guidelines addressing PA in populations beyond breast cancer. Also, most guidelines were not focused specifically on PA or exercise, but on broader topics such as symptom management or survivorship care. Many guidelines on survivorship care were primarily of relevance to individuals within the post-treatment phase of the cancer care continuum, demonstrating the need to develop tailored guidelines for people during active cancer treatment or living with advanced disease. Many guidelines were relevant to individuals presenting with certain cancer-related symptoms, such as fatigue or pain, supporting the shift towards impairment-driven rehabilitation in cancer care [52]. However, these findings highlight research gaps on the role of PA in the management of other cancer-related issues, such as depression and lymphedema.

### Overall quality of guidelines

Although the overall quality of the guidelines varied tremendously, several guidelines of acceptable quality were identified to direct stakeholders on targeted PA recommendations for a range of cancer populations. More than half of the guidelines met the quality threshold with domain scores of >60% for rigour of development and at least two other domains. These findings assist stakeholders in identifying well developed and presented guidelines for use in clinical and research practice. Of these, only one guideline by Segal et al. (2017) [36] focused solely on the topic of exercise for the general cancer population.

### Quality domains

The strongest domains in the included guidelines were related to scope and purpose and clarity of presentation. Lower quality scores were given for the domain of applicability. Our average AGREE II domain scores were similar to those reported in the 2012 Canadian Partnership Against Cancer (CPAC) Cancer Practice Guidelines Status Report for 10 guidelines in the survivorship, recovery and rehabilitation component [53]. These general findings were also shown in a comprehensive appraisal conducted by Harris et al. (2012) of a different set of guidelines on the rehabilitation management of various impairments in patients with breast cancer [11]. The AGREE II domain scores presented in our review were slightly higher, but followed the same pattern as the scores recently reported in the CPAC Cancer Guidelines Database [54] for five of the same guidelines identified in our review [41, 44, 45, 47, 50]. Additional guidelines were evaluated within this directory but different versions were used for their appraisals from those included in our review. For example, we used the 2017 update of the guidelines on integrative therapies for breast cancer by Greenlee et al. [39] while the Cancer Guidelines Database reported on the 2014 version [38]. Furthermore, we appraised guidelines published as peer-reviewed journal articles while the Cancer Guidelines Database sometimes referred to web-based organizational documents. This was the case for the guidelines on cancer-related fatigue by Berger et al, (2015) [25] and Howell et al. (2013) [33], on survivorship by Denlinger et al. (2014) [27–32] and on exercise for people with cancer by Segal et al. (2017) [36]. Despite these differences, their results were similar to ours in that the guidelines frequently scored lowest in applicability and moderately on stakeholder involvement, rigour of development, clarity of presentation and editorial independence.

An important area requiring considerable improvement among most included guidelines was the applicability and implementation of PA recommendations to practice for people with cancer. Specific challenges in considering resource implications and identifying auditing and monitoring criteria were noted in this review. In the application of PA programs for individuals with cancer, there are knowledge gaps in the areas of identifying optimal exercise parameters, timing and population type through well-designed randomized trials, assessing program cost-effectiveness and developing appropriate implementation strategies [12, 13]. Recently developed guidelines with acceptable scores in this domain (≥60%) [24, 27–32, 47] present examples of strategies to implement recommendations, such as assessment tools for patients and training programs for health professionals. Relevant information on pre-exercise assessments, cancer- and treatment-specific precautions to activity, and real-life examples of activities, was also provided in several guidelines. Further research is required to identify effective knowledge translation strategies that may facilitate the applicability of these guidelines to clinical practice.

Moderate quality scores were found for the domains of rigour of development, stakeholder involvement and editorial independence. Related to the rigour of development, adequate information was lacking in several guidelines on the literature search strategies and article selection criteria, the methods used to describe and grade the evidence, as well as the detailed process to formulate the recommendations. Many guidelines included in our analysis did not report on a formal external review of the guidelines. This process can help provide a thorough, comprehensive and unbiased evaluation of the recommendations by individuals who have clinical or research expertise in the area or who may benefit from use of the guidelines. Another important consideration is the procedure to update evidence-based guidelines. For example, the NCCN has released a 2016 update to the 2014 survivorship guidelines included in this review [55]. Given the rapid emergence of evidence in this evolving field of research, continuous review and periodic updating of the recommendations are necessary to ensure target users are informed of current evidence-based best practices [11, 14]. Using systematic and reproducible literature search methods, providing clear and adequate detail on how recommendations were formulated and modified, obtaining feedback from external reviewers, as well as regularly updating the guidelines are key strategies to enhance the rigour of guideline development in this area of research.

In terms of stakeholder involvement, there was a wide range of practices noted in the guidelines included in this review. Many guideline development teams were multidisciplinary, representing a variety of different professions and expertise areas. While the majority of the selected guidelines incorporated findings from observational studies on patient preferences and considered tailoring and individualization of PA programs, few reported on the inclusion of a patient representative on their team. Exploring and integrating the views and preferences of members of the target population, people diagnosed with cancer, is valuable in the process of developing guidelines for this population. This helps confirm that the recommendations are applicable and relevant for the target population. Although it would be beneficial to integrate patients throughout the guideline development process, involving patients at least within an external review process should be considered.

Regarding editorial independence, several recent guidelines reported extensively on funding sources among individual members of the guideline development teams. However, specific conflicts of interest and their potential influence on the development and content of the guideline were not always adequately addressed. Potential conflicts may be less of a concern in PA research compared to other fields but are still important to document as part of standard practice in the development of guidelines.

The clarity of presentation for the recommendations was appropriate in many guidelines reviewed. A particularly noteworthy example of guidelines for individuals with cancer where the PA recommendations and other relevant information were easily identifiable and clearly presented include the survivorship guidelines developed by the NCCN [27–32]. This aspect is important for patients, caregivers and even clinicians who may not have the time, knowledge or skills to search through lengthy, detailed documents for pertinent information.

An area of strength in the guidelines included in this review was the detailed overview of the scope and purpose of the recommendations. Most guidelines clearly defined their objectives and health questions, by identifying the general population, intervention and outcomes under study. However, in several cases, the target population to whom the recommendations applied was not always adequately described, with respect to age, cancer stage and treatment phase. This information would be valuable in ensuring appropriate application of the recommendations according to individual patient factors.

### Limitations

Limitations of our review include potential selection bias due to our limited inclusion criteria. Guidelines that were not published in scientific peer-reviewed journals, such as the National Institute for Health and Care Excellence (NICE) guidelines or recent versions of the NCCN guidelines, were excluded from our review. To ensure scientific rigour and minimize conflict of interest within the guidelines included, we developed our article selection criteria to exclude guidelines that had not undergone the extensive peer review process required for publication in scientific journals. Moreover, guidelines that did not contain recommendations on specific PA parameters were excluded. For example, several guidelines on the management of symptoms such as depression and sleep disorders [33, 56] suggested the benefits of general PA, but did not include precise recommendations. Moreover, we excluded guidelines including only recommendations related to therapeutic exercise (e.g. range of motion exercises [57] or pelvic floor therapy [58]) as these did not meet the criteria for our definition of PA and including these was beyond the scope of our study objectives and literature search strategy. Similarly, there may have been selection bias as guidelines that were not published in English were excluded from this review and this may explain the high proportion of guidelines developed in North America included in our review.

Limitations in our methodology include the use of one reviewer for the title scan due to the quantity of citations retrieved in our literature search. Moreover, as our review topic was specifically on PA and our literature search terms reflected this focus, we did not search guidelines on topics such as fatigue management or with specific cancer populations and thus, we may have missed other guidelines including PA recommendations. However, our review helps highlight methodological limitations and identify acceptable quality documents within a set of recently published guidelines that include PA recommendations for a range of cancer populations.

Additional limitations are related to the use of the AGREE II tool for the critical appraisal of the guidelines included in this review. The middle scores of the 7-point scale on the AGREE II tool have not been well defined for each item. There are no clear recommendations on interpreting domain and overall quality scores or set specific cut-off scores to differentiate between high and low-quality guidelines. Although based on previous guideline appraisals [20–22], the use of the 60% cut-off score is somewhat arbitrary. However, this tool permits a comprehensive assessment of guidelines and should be consulted in the development of future recommendations.

Finally, several guidelines highlighted the limitations of the primary research informing the recommendations. Many recommendations on PA were generic, focused on general activity, aerobic exercise and strengthening exercise, and often limited to the breast cancer population. These findings highlight the need for further research employing robust methods and exploring specific PA types and parameters, particularly in populations besides breast cancer [12, 13].

## Conclusions

- Several guidelines developed and published recently include PA recommendations for people diagnosed with cancer. These guidelines apply to a range of cancer types (mostly general or breast cancer) and cover various broad topics (e.g. survivorship care, fatigue management, integrative therapies).
- Based on the AGREE II criteria, strengths of current guidelines include the identification of scope and purpose, as well as the clarity of presentation. Improvement is needed in the applicability of guidelines.
- Although there are limitations in the primary research informing the recommendations, guidelines of acceptable quality exist to direct stakeholders on PA for the cancer population.
- Health professionals can play an important role in supporting people with cancer throughout the disease trajectory and benefit from access to well-developed and appropriate materials to interpret research knowledge on effective rehabilitation strategies, including PA.

## Supporting information

S1 Table Electronic database search strategy. (DOCX)

S2 Table Selection criteria for evidence-based guidelines. (DOCX)

S3 Table PRISMA checklist. (DOC)
