# Supplementary material for: Methodological quality of clinical practice guidelines with physical activity recommendations for people diagnosed with cancer: A systematic critical appraisal using the AGREE II tool
Source: PLoS One. 2019 Apr 10;14(4):e0214846. doi: 10.1371/journal.pone.0214846 (PMC6457630; doi:10.1371/journal.pone.0214846)
Supplement: S1 Table — (DOCX) [file pone.0214846.s001.docx]

**S1 Table. Electronic database search strategy**

| **Database** | **Search strategy** |
| --- | --- |
| **MEDLINE (Ovid)** | 1. exp Neoplasms/  2. (cancer* or neoplas* or carcinom* or adenocarcinom* or malignan* or tumor* or tumour*).tw.  3. 1 or 2  4. exp Exercise/  5. exp Exercise Therapy/  6. exp Physical Fitness/  7. exp Rehabilitation/  8. exercis*.tw.  9. (physical* adj4 activ*).tw.  10. rehab*.tw.  11. 4 or 5 or 6 or 7 or 8 or 9 or 10  12. exp Practice Guideline/  13. (recommendation* or guideline*).tw.  14. 12 or 13  15. 3 and 11 and 14  16. limit 15 to yr="2011 -Current" |
| **EMBASE (Ovid)** | 1. exp neoplasm/  2. (cancer* or neoplas* or carcinom* or adenocarcinom* or malignan* or tumor* or tumour*).tw.  3. 1 or 2  4. exp exercise/  5. exp kinesiotherapy/  6. exp physical activity/  7. exp fitness/  8. exp rehabilitation/  9. exp cancer rehabilitation/  10. exercis*.tw.  11. (physical* adj4 activ*).tw.  12. rehab*.tw.  13. 4 or 5 or 6 or 7 or 8 or 9 or 10 or 11 or 12  14. exp practice guideline/  15. (recommendation* or guideline*).tw.  16. 14 or 15  17. 3 and 13 and 16  18. limit 16 to yr="2011 -Current" |
| **CINAHL** | S15. Limited to publication date 2011-2017  S14. (S3 AND S12 AND S15)  S15. S13 OR S14  S14. TI ( (recommendation* or guideline*) ) OR AB ( (recommendation* or guideline*) )  S13. (MH "Practice Guidelines")  S12. S4 OR S5 OR S6 OR S7 OR S8 OR S9 OR S10 or S11  S11. TI (rehab*) OR AB (rehab*)  S10. TI (physical* N4 activ*) OR AB (physical* N4 activ*)  S9. TI exercis* OR AB exercis*  S8. (MH “Rehabilitation+”)  S7. (MH "Physical Fitness+")  S6. (MH "Physical Activity")  S5. (MH "Therapeutic Exercise+")  S4. (MH "Exercise+")  S3. S1 OR S2  S2. TI ( (cancer* or neoplas* or carcinom* or adenocarcinom* or malignan* or tumor* or tumour*) ) OR AB ( (cancer* or neoplas* or carcinom* or adenocarcinom* or malignan* or tumor* or tumour*) )  S1. (MH "Neoplasms+") |
| **PEDro** | Abstract & Title: “cancer”  Method: practice guidelines |
