# Supplementary material for: Methodological quality of clinical practice guidelines with physical activity recommendations for people diagnosed with cancer: A systematic critical appraisal using the AGREE II tool
Source: PLoS One. 2019 Apr 10;14(4):e0214846. doi: 10.1371/journal.pone.0214846 (PMC6457630; doi:10.1371/journal.pone.0214846)
Supplement: S2 Table — (DOCX) [file pone.0214846.s002.docx]

**S2 Table. Selection criteria for evidence-based guidelines**

| **Inclusion Criteria** | **Rationale for Selection Criteria** | **Exclusion Criteria** |
| --- | --- | --- |
| 1. Systematically developed guidelines, including a review of literature and clearly identified recommendations | 1. Guidelines should be evidence-based and provide clear recommendations for use in clinical practice | 1. Guidelines with no literature review or no clearly identified recommendations |
| 1. Guidelines published in peer-reviewed scientific journals | 1. Guidelines should be part of scientific literature | 1. Guidelines not published in peer-reviewed scientific journals |
| 1. Guidelines with reference list included | 1. Guidelines should be based on published scientific evidence | 1. Guidelines with no reference list |
| 1. Guidelines published in English | 1. Adequate knowledge of language required for review and appraisal of guidelines | 1. Guidelines published in other languages besides English |
| 1. Guidelines with full text available | 1. Full text required for complete review and appraisal of guideline | 1. Guidelines with no full text available |
| 1. Guidelines with recent date of publication (January 2012-June 2017), including updated guidelines | 1. Guideline should be current and updated based on recent literature | 1. Guidelines with older date of publication (< 2012) with no recent update |
| 1. Guidelines focused on adults with diagnosis of cancer (any type, stage or treatment phase) | 1. Recommendations may differ for pediatric or adolescent cancers, healthy population, and other conditions | 1. Guidelines focused on pediatric or adolescent cancer populations, cancer prevention or non-cancer diagnoses |
| 1. Guidelines including at least one recommendation related to physical activity or physical exercise parameter (including frequency, intensity, duration and/or type) | 1. Review focus is on physical activity, defined as “any bodily movement produced by skeletal muscles that results in energy expenditure” ([5](#_ENREF_5)) | 1. Guidelines focused on pharmaceutical, surgical or therapeutic (including rehabilitation) management of cancer or assessment related to exercise (risk assessment, exercise testing) |
